# Contribution of smoking change to 45-year trend in prevalence of chronic bronchitis in Finland

**DOI:** 10.1177/14034948221104351

**Published:** 2022-06-19

**Authors:** Ville A Vartiainen, Pekka jousilahti, Tiina Laatikainen, Erkki Vartiainen

**Affiliations:** 1 Department of Public Health and Welfare, Finnish institute for Health and Welfare, Helsinki, Finland; 2 Individualized Drug Therapy Research Program, University of Helsinki, Helsinki, Finland; 3 Department of Pulmonary Medicine, Helsinki University Hospital, Helsinki, Finland; 4 Institute of Public Health and Clinical Nutrition, University of Eastern Finland, Helsinki, Finland; 5 Joint Municipal Authority for North Karelia Social and Health Care (Siun Sote), Helsinki, Finland

**Keywords:** Tobacco smoking, chronic bronchitis, epidemiology

## Abstract

**Aims:**

Tobacco smoking has been identified as the most important risk factor of chronic bronchitis. The aim of this study was to assess the contribution of smoking to the trends in prevalence of chronic bronchitis among men and women in Finland.

**Methods:**

For this purpose, we analysed questionnaires included in national FINRISK and FinHealth studies conducted between 1972 and 2017 in 5-year intervals. A total of 26,475 men and 28,684 women aged 30–59 years were included in the analysis. In addition to smoking, age and socioeconomic status were used as risk factors in the logistic regression model.

**Results:**

Smoking in Finland has declined from 51% to 23% in men between 1972 and 2017. In women, it increased from 11% in 1972 to 23% in 2002, with a following decrease to 16% in 2017. The prevalence of chronic bronchitis has generally followed the trend of smoking. The population attributable risk was 60% in men and 49% in women. A decrease in chronic bronchitis was observed in male never-smokers.

**Conclusions:**

**Smoking is currently declining in Finland in both men and women. As result, the prevalence of chronic bronchitis is declining and it is approaching baseline independent of smoking. The decrease in never-smokers has yet to be explained.**

## Introduction

Chronic bronchitis (CB) is a condition that affects both the individual and society. It has been shown to reduce quality of life and increase respiratory exacerbations independently from other respiratory conditions [1]. It has also been shown to be associated with increased respiratory, cardiovascular and all-cause mortality [2–5]. From society’s perspective it is associated with increased use of health services, hospitalisation and healthcare costs [2, 6]. Many risk factors have been identified. There is an ongoing discussion on the role of air pollutants and fine particles on incident respiratory diseases including CB [7]. An association of CB with exposure to fine particles has been shown for particles with an aerodynamical diameter of less than 2.5 µm and less than 10 µm [8, 9].

The most important risk factor for CB is tobacco smoking, but also electronic cigarettes have been shown to induce a risk independently from conventional smoking [10–12]. Finland has had a very active tobacco control policy. The first major effort to reduce smoking was started in an eastern province in Finland called North Karelia, which had the highest coronary mortality in the world in 1972 [13]. Finland was one of the first countries in the world to introduce anti-smoking legislation. The Tobacco Act came into force in 1976 including an advertisement ban, a ban on selling tobacco to under 16-year-olds, mandatory health warnings on cigarettes packs, restrictions of smoking in schools and public places, and a requirement of 0.5% of revenue from the excise duty on tobacco to be used for work in reducing smoking. Later, in 1992, hidden advertisements were forbidden and the minimum purchase age for tobacco was raised to 18 years. In 1995, all working places had to be smoke free. In 2007, restaurants became smoke free, with a transition period to 2009. From 2012 it has been prohibited to have tobacco products visible in premises of sales, and in 2016 it became illegal to smoke in a car where under 15-year-old children are present. The prohibition on visibility of tobacco-related products was extended to, for example, smoking accessories and electronic cigarettes in 2017 [14].

Earlier a 25-year trend of CB in the 25–64 year age group has been published from this data set from 1982 to 2007 [2]. The aim of this study was to assess the contribution of smoking to 45-year trends from 1972 to 2017 in the prevalence of CB among 30–59-year-old men and women in Finland and to assess how well the previous results are generalisable to the longer time span.

## Methods

Methods of data collection have been described in detail elsewhere [2, 15]. Cardiovascular risk factor surveys were initially started for the evaluation of the North Karelia project in North Karelia and in the reference Kuopio province [13]. Later in the National FINRISK Study the content of surveys also included other non-communicable diseases. Survey areas were added better to represent all Finland. Nine cross-sectional risk factor surveys (FINRISK 1972–2012 and FinHealth 2017) were conducted in Finland every 5 years by the National Public Health Institute and its successor the Finnish Institute for Health and Welfare. Independent population surveys were conducted in five areas: provinces North Karelia and Kuopio since 1972, south-west Finland since 1983, in Helsinki capital area since 1992 and Oulu province since 1997. Random samples were drawn from the population register. The sampling was stratified according to sex and 10-year age group, and was modified to be compliant with the protocol of the World Health Organization’s MONICA project and recommendations of the European Health Risk Monitoring project on their introduction. The sampling procedure was different in 2017 but comparable with the previous surveys. The present study includes 26,475 men and 28,684 women aged 30–59 years. Participation has gradually declined from 91% to 63% and increased in 2017 to 65%. Fine particle emissions and household wood burning were used as proxies for fine particle exposure, as reported by Statistics Finland. The fine particle exposure proxies were available only as national level data and, therefore, it was not possible to assign it on individual or regional level.

CB was defined in subjects by a positive response to the same standard question ‘Do you bring up phlegm on most days or nights for at least as much as three months each year’ in a self-administered questionnaire. Smoking was assessed in the surveys by using a set of standardised questions in a self-administered questionnaire. Based on these responses the participants were classified into three categories: current smokers had smoked regularly for at least one year and had smoked during the preceding month. Ex-smokers had smoked regularly but had stopped smoking no later than a month ago. Never-smokers had never smoked regularly. As there was no significant difference in the prevalence of CB between ex-smokers and never-smokers these categories were pooled for most of the analysis. In the classification any form of tobacco smoking was defined as smoking. The number of factory-made cigarettes, self-enrolled cigarettes, pipe smoking times, and cigars were calculated as smoking times in a day. The number of regular smoking years was asked in the questionnaire [16].

The number of school years was asked in a questionnaire. Educational tertiles were used in the analyses because the school system has changed dramatically during the decades. The oldest participants in the 1972 survey were born in 1913 and the youngest in 2017 was born in 1987. Educational tertiles were calculated separately for each 5-year birth cohort born between 1913 and 1987. Raw prevalences are reported in the tables because age adjustment did not change any of the prevalences. The chi-square test was used for categorial variables and analyses of variance for continuing variables. Logistic regression models were run separately for men and women. The year of examination, age, school years tertiles, number of smoking times in a day, and number of smoking years, fine particle concentrations, and household wood burning were treated as continuous variables, while survey area was treated as a categorical covariate. In the final model survey area and proxies for fine particle exposure were left out of the model due to lack of significance. The attributable risk was defined as [17]:

(1) $$\mathrm{AR}=\frac{{\mathrm{RR}}_{e}-{\mathrm{RR}}_{u}}{{\mathrm{RR}}_{e}}$$

where AR is the attributable risk, RR_*e*_ is the relative risk of the exposed and RR_*u*_ is the relative risk of the unexposed. There was no clear difference between never-smokers and ex-smokers in terms of CB symptoms. Therefore, for the AR analysis current smokers are defined as exposed, and unexposed are those not currently smoking. All statistics were performed using IBM SPSS Statistics 27 release 27.0.0.0 for Microsoft Windows.

Due to the long period of data collection, the ethical approval process and participant informed consent vary, and were done according to ethical rules and Finnish legislation during the time of each study. The study was conducted following the rules of the Declaration of Helsinki on medical ethics.

## Results

**Figure 1.**
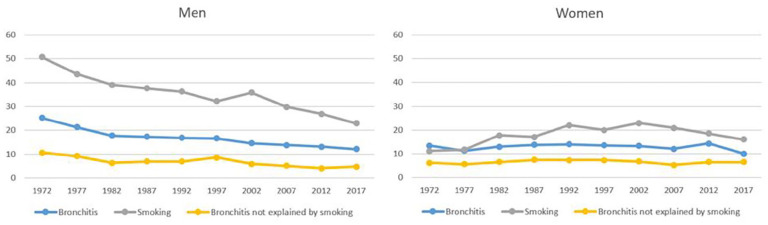
Prevalence of chronic bronchitis (CB), smoking and fraction of CB not explained by smoking according to attributable risk. The prevalence of CB not explained by smoking is defined as (100-AR) × prevalence, where AR is the attributable risk to smoking.

In men, smoking declined from 50.6% in 1972 to 23.0% in 2017 (Figure 1). The main reason for the decline in smoking was an increase in never-smokers from 28.1% to 49.6%, but the proportion of ex-smokers increased as well. In women, the development was different. In 1972, smoking in woman was 11.2%, increased to 22.9% in 2002 and started to decline after that, being 16.1% in 2017. The proportion of never-smokers has declined and ex-smokers increased. The number of smoking times in a day among current smoking men has declined from 18 in 1972 to 12 in 2017, and among women an increase from 10 to 13 was observed in 1992 and a decrease to eight in 2017.

In men, the prevalence of CB declined from 25.2% in 1972 to 12.2% in 2017. A statistically significant decreasing trend was seen in all smoking categories: among never-smokers the decline was from 13.1% to 9.8%, among ex-smoker from 17.5% to 8.0%, and among smokers from 35.2% to 24.4% (Table I). In women CB increased in the first 20 years and then started to decline. In different smoking categories there were no statistically significant changes. There was significantly more fluctuation in the prevalence of CB in women by examination year when compared with men. In most of the survey years, the prevalence of bronchitis was quite similar among never and ex-smokers. CB was found to be more prevalent in smoking men than in smoking women. In the non-smoking population, the prevalence was similar. The attributable risk of smoking remained roughly similar between the examination years.

**Table I.** Prevalence of chronic bronchitis (% (*n*)) by smoking status, number of smoking times in a day among current smokers, and attributable risk (%) of smoking by sex.

Men	1972	1977	1982	1987	1992	1997	2002	2007	2012	2017	Total	*P* value
Never-smoker % (*n*)	13.1 (174)	13.0 (173)	10.3 (120)	10.0 (81)	10.1 (83)	13.5 (143)	8.5 (85)	8.7 (70)	7.6 (61)	9.8 (34)	10.8 (1024)	<0.001
Ex-smoker % (*n*)	17.5 (176)	14.2 (180)	10.8 (104)	12.5 (75)	12.7 (73)	12.0 (82)	12.3 (71)	11.0 (52)	9.6 (42)	8.0 (15)	12.9 (870)	<0.001
Current smoker %(n)	35.2 (837)	31.4 (663)	29.2 (397)	27.6 (233)	26.9 (213)	24.8 (205)	23.4 (206)	24.2 (128)	26.5 (119)	24.4 (40)	29.4 (3011)	<0.001
Total % (*n*)	25.2 (1187)	21.4 (986)	17.8 (621)	17.3 (389)	16.9 (369)	16.7 (430)	14.7 (362)	13.9 (250)	13.2 (222)	12.3 (89)	18.5 (4905)	<0.001
Number of smoking times in a day *n* (*n*)	18 (2380)	19 (2050)	19 (1395)	19 (880)	19 (792)	18 (833)	17 (892)	17 (552)	16 (461)	12 (226)	18 (10,460)	<0.001
Attributable risk %	57.4	56.7	64.0	59.8	58.4	48.0	59.5	62.2	68.7	59.9	60.2	
Women	1972	1977	1982	1987	1992	1997	2002	2007	2012	2017	Total	*P* value
Never-smoker % (*n*)	11.8 (516)	10.3 (418)	11.6 (288)	12.4 (213)	12.2 (186)	12.2 (218)	11.7 (197)	10.4 (125)	11.8 (141)	9.5 (45)	11.5 (2347)	0.18
Ex-smoker (%)	15.0 (20)	7.7 (22)	7.2 (22)	10.2 (30)	9.2 (31)	9.3 (46)	8.5 (42)	8.8 (40)	11.9 (48)	8.8 (15)	9.4 (316)	0.23
Current smoker (%)	25.8 (147)	20.0 (115)	22.1 (133)	22.2 (91)	22.4 (117)	21.4 (122)	20.9 (135)	20.4 (89)	25.9 (94)	14.3 (18)	22.0 (1061)	0.25
Total (%)	13.5 (683)	11.3 (555)	13.0 (443)	13.8 (334)	14.0 (334)	13.6 (386)	13.3 (374)	12.1 (254)	14.4 (283)	11.3(88)	13.0 (3734)	0.02
Number of smoking times in a day *n* (*n*)	10 (570)	12 (589)	13 (630)	12 (433)	13 (524)	13 (576)	12 (654)	12 (446)	12 (367)	8 (175)	12 (4954)	<0.001
Attributable risk (%)	53.9	49.5	49.8	45.5	47.8	45.8	48.5	55.6	54.4	35.0	49.1	

Logistic regression models were calculated for men and women separately to predict CB (Table II). Among men age, year of the examination, current smoking, number of smoking times in a day and number of smoking years significantly predicted CB. Proxies for small particle exposure were not a significant factor and were left out of the model. The survey area was used as a categorical covariate in the models. In women age, education and smoking times in a day statistically significantly predicted CB. If the smoking times a day and the number of smoking years were left out from the models, the odds ratio for current smoking was 2.8 in men and 2.2 in women. Self-reported asthma was also a significant risk factor, but as it did not affect the magnitude or significance of other factors, it was also left out of the models.

**Table II.** Logistic regression models for chronic bronchitis.

Men	β	OR	*P* value	95% Confidence interval	
Year of examination	–0.069	0.934	<0.001	0.919	0.948
Age (years)	0.025	1.025	<0.001	1.020	1.030
Educational tertile (1–3)	–0.178	0.837	<0.001	0.802	0.873
Smoking (yes/no)	0.324	1.382	<0.001	1.229	1.554
Number of smoking times in a day (*n*)	0.030	1.030	<0.001	1.026	1.035
Number of smoking years (*n*)	0.015	1.015	<0.001	1.011	1.019
Year of examination	–0.001	0.999	0.874	0.993	1.014
Women	β	OR	*P* value	95% Confidence interval	
Age (years)	0.026	1.026	<0.001	1.021	1.030
Educational tertile (1–3)	–0.150	0.861	<0.001	0.824	0.900
Smoking (yes/no)	0.049	1.050	0.548	0.895	1.233
Number of smoking times in a day (*n*)	0.063	1.065	<0.001	1.055	1.075
Number of smoking years (*n*)	0.002	1.002	0.488	0.996	1.008

OR: odds ratio.

## Discussion

Tobacco smoking has been regarded as the most important cause of CB. However, in our cohort the declining tobacco smoking did not fully explain the change in prevalence. In men, the decline was also found in never-smokers, and bronchitis not explained by smoking halved between 1972 and 2017 (see Figure 1). In women, the trend is not as apparent, but the small decline in prevalence in never-smokers was seen. There was no difference between never-smokers and ex-smokers one month after cessation, indicating that the symptoms are alleviated already during the first few weeks. In the logistic regression model in which only the current smoking was included, the odds ratio for CB was slightly higher in men than in women, which can be explained by greater smoking times in a day in men. To model environmental exposures on population level we included fine particulate concentrations in air when data were available, but no association was found to the symptoms. As measurements for particles were only available from 1991, we tried to use small scale wood burning for house heating as a proxy for the whole time period, but no association was found for that either.

In contrast to the findings of Pelkonen et al. [18] age did not have an effect on the decline of CB during the follow-up. In Pelkonen et al. [18] the effect was most prominent in the oldest age group, which was largely excluded from our study. In a population sample from the Finnish capital Helsinki, Kainu et al. [19] found the prevalence of CB to be stagnant between 1996 and 2006 in contrast to our results, which show a small but detectable decline in prevalence during the same time period. In a Finnish twin study ex-smokers were more likely to have symptoms of CB than never-smokers [20]. The trend of prevalence seems to vary in different populations. Lindström et al. [21] showed a significant difference in prevalence between northern Finland and northern Sweden despite similar geographical and socioeconomic factors. In a prospective study in Saskatchewan, Pahwa et al. [22] found the prevalence of CB to be 6.4% and 5.3% in 2010 and 12.1% and 9.2% in 2014 for non-farm and farm residents, respectively, despite a decrease in current smokers. In the RHINE study, conducted in 1999–2001, the prevalence was 5.4% in northern Europe, which is markedly lower than in our study [23]. Axelsson et al. [24] found the prevalence of CB to be 15% in 1990 and 7.2% in 2013 in west Sweden. The authors found CB to be associated with smoking, age and low socioeconomic status [24]. A population-based study in five Colombian cities found the prevalence of CB to be 5.5%, with risk factors being current smoking, male sex, age of 64 years or older, low education and environmental exposures [25]. In an Italian study, the prevalence was stagnant between 1998/2000 and 2007/2010, being 12.5% and 12.6%, respectively, despite a decrease in current smokers. CB was associated with current smoking and female sex as well as asthma and allergic rhinitis [26]. These differing results are likely to be attributed to environmental exposures not adequately captured in the studies. However, there are studies showing an association with environmental exposures such as fine particles and nitric dioxide to CB [8, 9]. There is also evidence of CB associating with biomass fuels, dusts, chemical fumes and smoky domestic fuels [27]. In this work the proxies for environmental exposures were at the population level only, and as such they do not necessarily reflect the exposure at an individual level.

Because of large differences in socioeconomic status, culture, climate and smoking in the different countries, the study results are not likely to be comparable. Despite the varying trend, smoking and socioeconomic status seem to be appearing as risk factors everywhere. One of the weaknesses of this and other studies is that there is no objective marker for CB. As it is based on surveys of symptoms, it is prone to different interpretations, especially across cultural borders. Also, there is variation in the questionnaires and definitions, which makes the comparison of the studies difficult. In our study, the study regions have changed over time and the sampling has not been nationally representative. This adds a degree of error in the temporal comparisons. The participation rate has declined over time, making the current analysis more susceptible to selection bias. Our results are in line with the previous studies carried out in Finland, which is not surprising as a large proportion of the subjects are shared between the studies [2].

The tobacco epidemic in Finland is following the model described by Lopez et al. [28]. In stage I, smoking is uncommon in both men and women and it is not captured in this study. In 1972, Finland was clearly in stage II smoking, in which smoking was common in men but relatively rare among women. During the Second World War, free cigarettes were given to the soldiers for 5 years, and as a result a large majority of the male population was smoking afterwards. Around the 1980s Finland moved to stage III, in which smoking in men declined while it increased in women. In the model of Lopez et al. [28] female smoking in stage III typically increases to 35–45%. In Finland, it plateaued under 25% during the 1990s and never increased to the higher levels seen in many other countries. In birth cohort analyses in women, the increase in the onset of smoking ended in the birth cohort born in the 1950s. The main reason was that in Finland the first Tobacco Act was adopted in 1976 when these women were at age of typical smoking onset in Finnish society. After 2002, Finland entered stage IV in which smoking is declining in both men and women and is approaching a state in which smoking is rare in both. The latest prevalence of smoking is 16% in men and 12% in women in the 15- to 74-year age group [29]. As the Lopez model does not cover a situation in which smoking plateaus in very low levels, we are proposing a new stage to be added: smoking is rare or non-existent in both men and women.

Passive smoking significantly decreased during the study period due to the changes in tobacco legislation [30]. Between 1992 and 2012 exposure to environmental tobacco smoke decreased from 25% to 5%. As environmental tobacco smoke is associated with CB it is likely to play a role in the declining prevalence of CB not explained by the decline in smoking [31]. There has been a significant shift of smoking habits in the workplace. Previously it was common that smoking was allowed everywhere. From there, employers have gradually changed their policies first to limit smoking to specified places and later prohibiting it in their property altogether. There has also been a shift from agriculture and industrial society to information society, and thus the typical work has changed from manual labour to office work. For example, welding fumes have been shown to be associated with CB [23], and the shift in the working environment has likely decreased similar exposures of the working age population. The general hygiene level, vaccinations and modern treatments have probably decreased respiratory infections, especially in childhood [32, 33]. Also, nutrition and living standards have increased during the decades contributing to the decrease seen in respiratory infections.

## Conclusions

The prevalence of CB has been declining since 1972. Smoking has also been declining in Finland, and it is evident that the country has entered stage IV in the Lopez model. The effects of Finland’s active tobacco policy were already seen in the 1970s when the increase in the onset of smoking in women was halted. The decrease in CB is partly explained by the decrease in smoking. However, a similar trend is seen in the never-smoker population also indicating that not all contributing factors are captured by the smoking status of the subject. In the end, the decline in tobacco smoking will lead to a change in the aetiology of CB when tobacco smoking is no longer the governing cause of the disease.

## Supplemental Material

sj-jpg-1-sjp-10.1177_14034948221104351 – Supplemental material for Contribution of smoking change to 45-year trend in prevalence of chronic bronchitis in Finland Click here for additional data file. Supplemental material, sj-jpg-1-sjp-10.1177_14034948221104351 for Contribution of smoking change to 45-year trend in prevalence of chronic bronchitis in Finland by Ville A Vartiainen, Pekka jousilahti, Tiina Laatikainen and Erkki Vartiainen in Scandinavian Journal of Public Health

sj-jpg-2-sjp-10.1177_14034948221104351 – Supplemental material for Contribution of smoking change to 45-year trend in prevalence of chronic bronchitis in Finland Click here for additional data file. Supplemental material, sj-jpg-2-sjp-10.1177_14034948221104351 for Contribution of smoking change to 45-year trend in prevalence of chronic bronchitis in Finland by Ville A Vartiainen, Pekka jousilahti, Tiina Laatikainen and Erkki Vartiainen in Scandinavian Journal of Public Health

## References

[bibr1-14034948221104351] Mejza F Gnatiuc L Buist AS , et al. Prevalence and burden of chronic bronchitis symptoms: results from the BOLD study. Eur Respir J 2017;50:1700621. DOI: 10.1183/13993003.00621-2017. 10.1183/13993003.00621-2017 PMC5699921 29167298

[bibr2-14034948221104351] Pelkonen M Notkola I-L Nissinen A , et al. Thirty-year cumulative incidence of chronic bronchitis and COPD in relation to 30-year pulmonary function and 40-year mortality: a follow-up in middle-aged rural men. Chest 2006;130:1129–1137. DOI: 10.1378/chest.130.4.1129. 17035447 10.1378/chest.130.4.1129

[bibr3-14034948221104351] Guerra S Sherrill DL Venker C , et al. Chronic bronchitis before age 50 years predicts incident airflow limitation and mortality risk. Thorax 2009;64:894–900. DOI: 10.1136/thx.2008.110619. 19581277 10.1136/thx.2008.110619 PMC4706745

[bibr4-14034948221104351] Lange P Parner J Prescott E , et al. Chronic bronchitis in an elderly population. Age Ageing 2003;32:636–642. DOI: 10.1093/ageing/afg108. 14600005 10.1093/ageing/afg108

[bibr5-14034948221104351] Jousilahti P Vartiainen E Tuomilehto J , et al. Symptoms of chronic bronchitis and the risk of coronary disease. Lancet (London, England) 1996;348:567–572. DOI: 10.1016/S0140-6736(96)02374-4. 8774568 10.1016/S0140-6736(96)02374-4

[bibr6-14034948221104351] Miravitlles M Murio C Guerrero T , et al. Costs of chronic bronchitis and COPD: a 1-year follow-up study. Chest 2003;123:784–791. https://pubmed.ncbi.nlm.nih.gov/12628879/ (accessed 21 May 2022). 12628879 10.1378/chest.123.3.784

[bibr7-14034948221104351] Thurston GD Balmes JR Garcia E , et al. Outdoor air pollution and new-onset airway disease: An official American Thoracic Society Workshop report. Ann Am Thorac Soc 2020;17:387–398. https://pubmed.ncbi.nlm.nih.gov/32233861/ (accessed 26 January 2021). 32233861 10.1513/AnnalsATS.202001-046ST PMC7175976

[bibr8-14034948221104351] Abbey DE Ostro BE Petersen F , et al. Chronic respiratory symptoms associated with estimated long-term ambient concentrations of fine particulates less than 2.5 microns in aerodynamic diameter (PM2.5) and other air pollutants. J Expo Anal Environ Epidemiol 5:137–159. http://www.ncbi.nlm.nih.gov/pubmed/7492903 (1995, accessed 7 June 2022). 7492903

[bibr9-14034948221104351] Hooper LG Young MT Keller JP , et al. Ambient air pollution and chronic bronchitis in a cohort of U.S. women. Environ Health Perspect 2018;126:027005. https://pubmed.ncbi.nlm.nih.gov/29410384/ (accessed 26 January 2021). 10.1289/EHP2199 PMC6066337 29410384

[bibr10-14034948221104351] Sethi JM Rochester CL . Smoking and chronic obstructive pulmonary disease. Clin Chest Med 2000;21:67–86; viii. DOI: 10.1016/s0272-5231(05)70008-3. 10763090 10.1016/s0272-5231(05)70008-3

[bibr11-14034948221104351] Eisner MD Anthonisen N Coultas D , et al. An official American Thoracic Society public policy statement: novel risk factors and the global burden of chronic obstructive pulmonary disease. Am J Respir Crit Care Med 2010;182:693–718. DOI: 10.1164/rccm.200811-1757ST. 20802169 10.1164/rccm.200811-1757ST

[bibr12-14034948221104351] Xie W Kathuria H Galiatsatos P , et al. Association of electronic cigarette use with incident respiratory conditions among us adults from 2013 to 2018. JAMA Netw Open 2020;3:e2020816. DOI: 10.1001/jamanetworkopen.2020.20816. 10.1001/jamanetworkopen.2020.20816 PMC7662143 33180127

[bibr13-14034948221104351] Puska P Bansilal S Narula J . The North Karelia Project: the spark that ignited the flame! Glob Heart 2016;11:171. https://globalheartjournal.com/article/10.1016/j.gheart.2016.05.001/ (accessed 21 May 2022). 10.1016/j.gheart.2016.05.001 27242082

[bibr14-14034948221104351] Finnish Tobacco Act 549/2016. https://www.finlex.fi/en/laki/kaannokset/2016/en20160549_20161374.pdf (accessed 21 May 2022).

[bibr15-14034948221104351] Borodulin K Tolonen H Jousilahti P , et al. Cohort profile: the National FINRISK Study. Int J Epidemiol 2018;47:696–696i. 29165699 10.1093/ije/dyx239

[bibr16-14034948221104351] Vartiainen E Puska P Koskela K , et al. Ten-year results of a community-based anti-smoking program (as part of the North Karelia Project in Finland. Health Educ Res 1986;1:175–184. https://academic.oup.com/her/article-lookup/doi/10.1093/her/1.3.175 (accessed 21 May 2022).

[bibr17-14034948221104351] Bhopal R . Concepts of Epidemiology. New York: Oxford University Press Inc., 2002, 209 pp.

[bibr18-14034948221104351] Pelkonen MK Notkola I-LK Laatikainen TK , et al. Twenty-five year trends in prevalence of chronic bronchitis and the trends in relation to smoking. Respir Med 2014;108:1633–1640. https://linkinghub.elsevier.com/retrieve/pii/S0954611114002893 (accessed 21 May 2022). 25195139 10.1016/j.rmed.2014.08.007

[bibr19-14034948221104351] Kainu A Pallasaho P Pietinalho A . No change in prevalence of symptoms of COPD between 1996 and 2006 in Finnish adults – a report from the FinEsS Helsinki Study. Eur Clin Respir J 2016;3:31780. https://www.tandfonline.com/doi/full/10.3402/ecrj.v3.31780 (accessed 21 May 2022). 27534614 10.3402/ecrj.v3.31780 PMC4989180

[bibr20-14034948221104351] Hukkinen M Korhonen T Broms U , et al. Long-term smoking behavior patterns predicting self-reported chronic bronchitis. COPD J Chronic Obstr Pulm Dis 2009;6:242–249. http://www.tandfonline.com/doi/full/10.1080/15412550903051781 (accessed 21 May 2022). 10.1080/15412550903051781 19811382

[bibr21-14034948221104351] Lindström M Jönsson E Kotaniemi J , et al. Smoking, respiratory symptoms, and diseases. Chest 2001;119:852–861. https://linkinghub.elsevier.com/retrieve/pii/S001236921551684X (accessed 21 May 2022). 11243968 10.1378/chest.119.3.852

[bibr22-14034948221104351] Pahwa P Rana M Amin K , et al. Incidence and longitudinal changes in prevalence of chronic bronchitis in farm and non-farm rural residents of Saskatchewan. J Occup Environ Med 2019;61:347–356. http://journals.lww.com/00043764-201904000-00012 (accessed 21 May 2022). 30789445 10.1097/JOM.0000000000001560

[bibr23-14034948221104351] Holm M Kim J-L Lillienberg L , et al. Incidence and prevalence of chronic bronchitis: impact of smoking and welding. The RHINE study. Int J Tuberc Lung Dis 2012;16:553–557. https://www.ingentaconnect.com/content/10.5588/ijtld.11.0288 (accessed 21 May 2022). 22325166 10.5588/ijtld.11.0288

[bibr24-14034948221104351] Axelsson M Ekerljung L Eriksson J , et al. Chronic bronchitis in West Sweden – a matter of smoking and social class. Eur Clin Respir J 2016;3:30319. https://www.tandfonline.com/doi/full/10.3402/ecrj.v3.30319 (accessed 21 May 2022). 27421832 10.3402/ecrj.v3.30319 PMC4947195

[bibr25-14034948221104351] Gonzalez-Garcia M Caballero A Jaramillo C , et al. Chronic bronchitis: high prevalence in never smokers and underdiagnosis – a population-based study in Colombia. Chron Respir Dis 2019;16:147997231876977. http://journals.sagepub.com/doi/10.1177/1479972318769771 (accessed 21 May 2022). 10.1177/1479972318769771 PMC6302977 29669432

[bibr26-14034948221104351] Accordini S Corsico AG Cerveri I , et al. Diverging trends of chronic bronchitis and smoking habits between 1998 and 2010. Respir Res 2013;14:16. DOI: 10.1186/1465-9921-14-16. 23394461 10.1186/1465-9921-14-16 PMC3574861

[bibr27-14034948221104351] Kim V Criner GJ . Chronic bronchitis and chronic obstructive pulmonary disease. Am J Respir Crit Care Med 2013;187:228–237. DOI: 10.1164/rccm.201210-1843CI. 23204254 10.1164/rccm.201210-1843CI PMC4951627

[bibr28-14034948221104351] Lopez AD Collishaw NE Piha T . A descriptive model of the cigarette epidemic in developed countries. Tob Control 1994;3:242–247. https://tobaccocontrol.bmj.com/lookup/doi/10.1136/tc.3.3.242 (accessed 21 May 2022).

[bibr29-14034948221104351] Statistics Finland. Tobacco statistics 2020, Tilastoraportti 38/2021. https://www.stat.fi/en/statistics/tupk (2021, accessed 7 June 2022).

[bibr30-14034948221104351] Jousilahti P Helakorpi S . Prevalence of exposure to environmental tobacco smoke at work and at home – 15-year trends in Finland. Scand J Work Environ Health 2002;28(Suppl.2):16–20. http://www.ncbi.nlm.nih.gov/pubmed/12058798 (accessed, 7 June 2022). 12058798

[bibr31-14034948221104351] Pelkonen MK Laatikainen TK Jousilahti P . The relation of environmental tobacco smoke (ETS) to chronic bronchitis and mortality over two decades. Respir Med 2019;154:34–39. https://linkinghub.elsevier.com/retrieve/pii/S0954611119301945 (accessed 21 May 2022). 31207539 10.1016/j.rmed.2019.06.006

[bibr32-14034948221104351] Azor-Martinez E Cobos-Carrascosa E Seijas-Vazquez ML , et al. Hand hygiene program decreases school absenteeism due to upper respiratory infections. J School Health 2016;86:873–881. http://doi.wiley.com/10.1111/josh.12454 (accessed 21 May 2022). 27866386 10.1111/josh.12454

[bibr33-14034948221104351] Sigurdsson S Kristinsson KG Erlendsdóttir H , et al. Decreased incidence of respiratory infections in children after vaccination with ten-valent pneumococcal vaccine. Pediatr Infect Dis J 2015;34:1385–1390. https://journals.lww.com/00006454-201512000-00023 (accessed 21 May 2022). 26780024 10.1097/INF.0000000000000899

