# Supplementary figures and images for: Contribution of smoking change to 45-year trend in prevalence of chronic bronchitis in Finland
Source: Scand J Public Health. 2022 Jun 19;51(8):1189–95. doi: 10.1177/14034948221104351 (PMC10642213; doi:10.1177/14034948221104351)

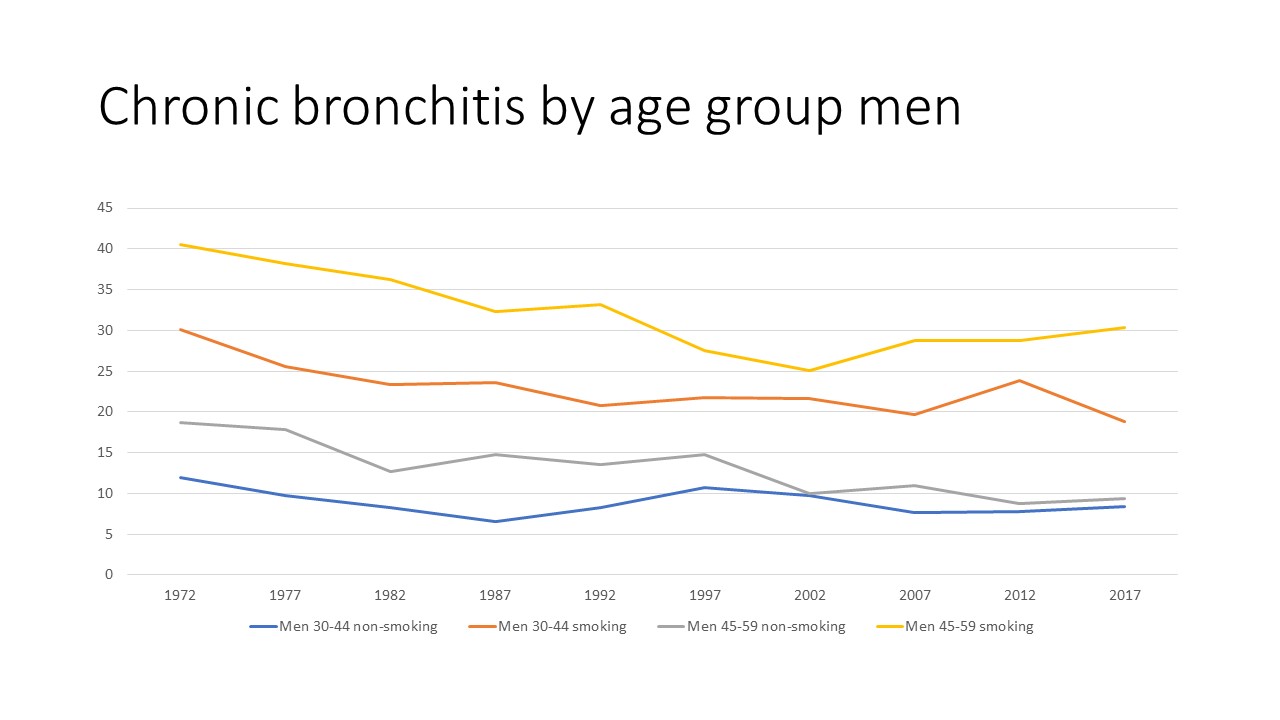

Supplement: sj-jpg-1-sjp-10.1177_14034948221104351 – Supplemental material for Contribution of smoking change to 45-year trend in prevalence of chronic bronchitis in Finland [file sj-jpg-1-sjp-10.1177_14034948221104351.JPG]

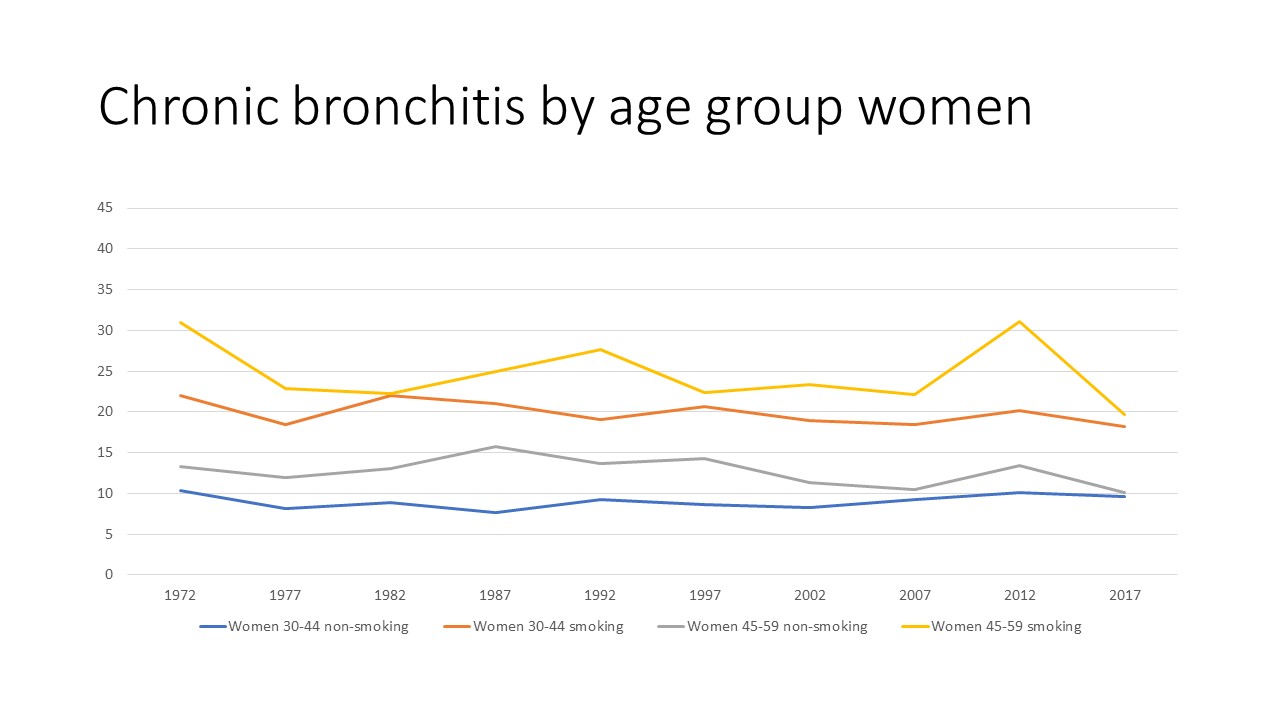

Supplement: sj-jpg-2-sjp-10.1177_14034948221104351 – Supplemental material for Contribution of smoking change to 45-year trend in prevalence of chronic bronchitis in Finland [file sj-jpg-2-sjp-10.1177_14034948221104351.JPG]
